# Well-Being and Dispositional Hope in a Sample of Portuguese Citizens: The Mediating Role of Mental Health

**DOI:** 10.3390/ejihpe14070140

**Published:** 2024-07-22

**Authors:** Maria João Velez, Helena A. Marujo, Zaida Charepe, Ana Querido, Carlos Laranjeira

**Affiliations:** 1 Department of Human Resources and Organizational Behavior, ISCTE-Instituto Universitário de Lisboa, Avenida das Forças Armadas, 1649-026 Lisboa, Portugal; maria.joao.velez@iscte-iul.pt; 2 Instituto Superior de Ciências Sociais e Políticas, Centro de Administração e Políticas Públicas (CAPP), Universidade de Lisboa, Rua Almerindo Lessa, 1300-663 Lisboa, Portugal; hmarujo@iscsp.ulisboa.pt; 3 Centre for Interdisciplinary Research in Health (CIIS), Universidade Católica Portuguesa, Palma de Cima, 1649-023 Lisboa, Portugal; zaidacharepe@ucp.pt; 4 Faculty of Health Sciences and Nursing, Universidade Católica Portuguesa, Palma de Cima, 1649-023 Lisboa, Portugal; 5 School of Health Sciences, Campus 2, Polytechnic University of Leiria, Morro do Lena, Alto do Vieiro, Apartado 4137, 2411-901 Leiria, Portugal; ana.querido@ipleiria.pt; 6 Centre for Innovative Care and Health Technology (ciTechCare), Campus 5, Polytechnic University of Leiria, Rua das Olhalvas, 2414-016 Leiria, Portugal; 7 Center for Health Technology and Services Research (CINTESIS), NursID, University of Porto, 4200-450 Porto, Portugal; 8 Comprehensive Health Research Centre (CHRC), University of Évora, 7000-801 Évora, Portugal

**Keywords:** well-being, mental health, dispositional hope, positive psychology

## Abstract

In our pursuit of a fulfilling and contented life, the study of well-being has emerged as a fundamental field of research. Higher levels of well-being are associated with better mental health outcomes. Individuals with better mental health might possess the personal resources necessary to set and pursue meaningful goals, maintain positive expectations, and overcome adversities. We aim to explore the positive relationship between well-being (hedonic, psychological, and social) and dispositional hope. We suggest that mental health acts as a mediator in this relationship, since improved mental health can create a conducive environment for the development and maintenance of dispositional hope. Data were collected using an e-survey through social media during the last quarter of 2022. The hypothesis of this study was tested using mediation analysis. The sample was composed of 471 participants (85.4% female) with a mean age of 47.72 ± 11.86 years. Participants were mainly workers (88.6%), followed by pensioners (6.8%), university students (2.5%), and unemployed (2.1%). Results revealed that well-being was positively and significantly associated with dispositional hope. Additionally, well-being presented a significant and positive relationship with mental health, which, in turn, also presented a significant and positive relationship with dispositional hope. Finally, using the Hayes process macro for SPSS, we found that mental health mediates the relationship between well-being and dispositional hope. Our findings reinforce the conceptual frameworks that consider well-being and mental health as key contributors to a resilient and optimistic mindset. Interventions that aim to cultivate positive affect, facilitate personal growth, and foster supportive social environments might help improve mental health outcomes.

## 1. Introduction

The study of well-being has become essential to understanding how to achieve a satisfying and contented life [1,2]. Indeed, as highlighted by Costanza et al. [3] and Judge and Kammeyer-Mueller [4], feeling good and discovering fulfillment and purpose (referred to as well-being) are increasingly important for individuals, organizations, and societies.

The tripartite model of well-being (including the hedonic, social, and psychological dimensions) expands our understanding of human happiness and satisfaction [5,6].

Hedonic well-being pertains to the subjective experience of pleasure, happiness, and overall life satisfaction. It involves the pursuit of positive emotions and the avoidance of negative ones [7,8]. While the importance of hedonic well-being has long been recognized, contemporary research delves deeper into the factors influencing this state [9]. Thus, hedonic well-being stems from and contributes to optimal functioning [10].

Social well-being underscores the significance of interpersonal relationships, social connections, and a sense of belonging within a community [11]. As social beings, our well-being is highly influenced by the quality of our relationships. This emphasizes the importance of exploring social support, networks, and integration [12]. Additionally, it helps identify the repercussions of social isolation and loneliness, emphasizing the importance of nurturing meaningful connections [13].

Psychological well-being focuses on self-awareness, personal growth, life purpose, autonomy, resilience and self-esteem, and coping with life’s challenges [14,15,16]. Moreover, studies have shown that individuals with higher levels of subjective well-being, life satisfaction, and positive affect tend to exhibit greater dispositional hope [17,18]. Enhanced psychological well-being enables individuals to approach challenges with optimism and persistence, fostering a lasting belief in their ability to pursue and achieve goals while maintaining a sense of agency and pathways to success (i.e., dispositional hope).

Indeed, how well-being and dispositional hope are expressed can be influenced by contextual and cultural factors. Further clarity is needed to understand how different cultures affect the patterns of expression of well-being and dispositional hope. Unlike most European countries, Portugal has a more collectivist cultural orientation, which means there are shared behaviors, assumptions, attitudes, values, historical background, and language groupings [19]. In this context, the standards established by the group take precedence over individual emotions and desires, and one’s sense of self is defined in relation to others [19].

**Figure 1:**
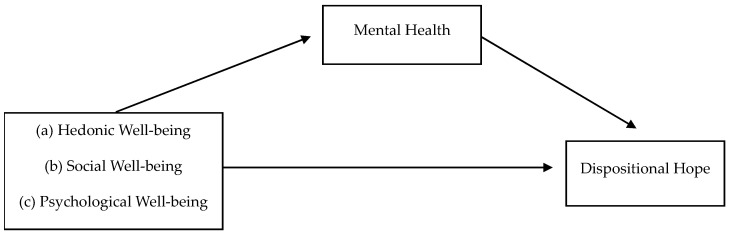
Hypothesized model of the relations between well-being and dispositional hope, with mental health playing a mediating role.

Therefore, the present research seeks to advance our understanding of the factors influencing mental health (in terms of the absence of anxiety symptoms) and dispositional hope by proposing a mechanism for the relationship between well-being and dispositional hope. Furthermore, to the best of our knowledge, no Portuguese study has explored these relationships. Figure 1 presents the conceptual model.

### 1.1. Well-Being and Dispositional Hope

The interaction between well-being and dispositional hope and how these constructs intertwine and impact each other has garnered considerable attention in the literature [20,21,22]. Dispositional hope refers to an individual’s inherent optimism and belief in their capacity to pursue and accomplish goals. It encompasses a sense of agency (the perception of being capable of acting) and pathway thinking (the identification of feasible routes to achieve desired outcomes) [23,24].

Research has indicated a positive relationship between dispositional hope and positive emotions, life satisfaction, and overall subjective well-being [25,26]. The experience of positive emotions and happiness fosters a hopeful mindset, enabling individuals to cherish and derive meaning from positive experiences [27,28]. Moreover, well-being cultivates adaptive coping strategies, increasing the likelihood of employing problem-solving techniques, seeking social support, and utilizing positive coping mechanisms when confronted with challenges or setbacks [21]. These adaptive coping strategies aid in stress management and mitigate negative emotions, facilitating a heightened sense of dispositional hope. Furthermore, well-being shapes one’s perception of life events and interpretation of circumstances. Individuals with greater well-being tend to engage in positive reappraisal, framing challenges as opportunities for growth and viewing setbacks as temporary and surmountable [29]. This optimistic outlook contributes to a heightened hedonic well-being and a more positive life perspective.

Previous research also indicates that dispositional hope plays a significant role in promoting social well-being and various facets of social functioning [30]. Social well-being encompasses the quality of an individual’s social relationships, social integration, and sense of belonging within a community. Previous research has consistently shown a positive relationship between social well-being and dispositional hope, i.e., individuals with greater social well-being tend to experience higher levels of dispositional hope [31,32,33,34]. Individuals engaging in positive and meaningful social interactions tend to develop a hopeful mindset, because establishing and maintaining supportive and fulfilling social connections contributes to positive affect, optimism, and resilience [21,35]. Additionally, proactive social engagement and active participation in social activities foster the development of adaptive coping strategies in the face of social challenges and setbacks, showcasing resilience and the ability to rebound from social difficulties [27].

In turn, psychological well-being encompasses elements such as positive emotions, life satisfaction, self-acceptance, personal growth, purpose in life, and positive relationships [36]. Individuals with higher psychological well-being exhibit greater dispositional hope due to their optimistic attitudes and resilience [27,30,37,38]. Indeed, the experience of positive emotions is linked to adaptive coping strategies and resilience in adversity, increasing the likelihood of employing problem-solving skills, seeking social support, and engaging in positive coping mechanisms when confronted with challenges [23,39]. This enhances the ability to navigate and overcome stressful situations, fostering a clearer sense of individual goals, values, and aspirations, thus contributing to a more fulfilling and satisfying life [23].

### 1.2. Well-Being and Mental Health

The relationship between well-being and mental health is intricate and multi-dimensional. Although used interchangeably, “well-being” and “mental health” represent distinct yet interconnected facets of an individual’s overall psychological state [40]. As previously noted, well-being encompasses a broad spectrum of factors that are important for a person’s overall quality of life and subjective experience, including hedonic well-being (such as experiencing positive emotions and life satisfaction), psychological well-being (such as self-acceptance, personal growth, and purpose in life), and social well-being (involving positive social relationships and a sense of belonging) [6,7]. In turn, mental health refers to an individual’s state of psychological well-being in terms of their emotional, cognitive, and behavioral functioning. It denotes the absence of mental disorders or illnesses and the presence of subjective well-being.

Research consistently underscores a robust relationship between well-being and mental health. Elevated levels of well-being typically correspond to improved mental health outcomes, including reduced levels of psychological distress, decreased risk of mental disorders, and enhanced overall mental well-being [41,42]. Conversely, diminished individual well-being across hedonic, psychological, and social domains can negatively affect mental health, potentially leading to mental disorders such as depression, anxiety, and substance abuse [43]. Therefore, endeavors to promote mental health and well-being require a holistic approach that addresses both the prevention and treatment of mental disorders and the promotion of positive psychological functioning and well-being.

In this sense, higher levels of psychological well-being are typically associated with improved mental health outcomes, including decreased psychological distress, reduced risk of mental disorders, and enhanced overall mental well-being [15,41,44]. Contrarily, poor mental health can significantly impact psychological well-being, i.e., mental disorders—such as depression, anxiety, and substance abuse—can impair various dimensions of psychological well-being, including emotional well-being, self-esteem, personal growth, and the ability to establish and maintain positive relationships [45,46]. However, distinguishing between mental health and psychological well-being is important; while mental health refers to the absence of mental disorders, psychological well-being emphasizes positive psychological functioning and a sense of purpose in life [44].

Evidence demonstrates that higher levels of hedonic well-being are associated with improved mental health outcomes, including reduced psychological distress, decreased risk of mental disorders, and enhanced overall mental well-being [41,47]. Positive emotions and experiences associated with hedonic well-being protect mental health, since they buffer against stress, enhance resilience, and promote adaptive coping strategies [48]. In turn, low levels of hedonic well-being, characterized by a lack of positive emotions and frequent experiences of negative affect, are linked to an increased risk of mental health problems, such as depression and anxiety [49,50].

Similarly, the relationship between social well-being and mental health is an important area of research that explores how social factors and relationships influence an individual’s mental health outcomes. Higher levels of social well-being are typically associated with improved mental health outcomes, including reduced psychological distress, decreased risk of mental disorders, and enhanced overall mental well-being [41,51]. Strong social connections provide emotional support, practical assistance, and a sense of belonging. Therefore, they buffer against stress, enhance coping mechanisms, and promote psychological resilience [52,53]. Conversely, poor social well-being—characterized by social isolation, loneliness, or relationship conflict—is associated with an increased risk of mental health problems, such as depression, anxiety, and substance abuse [54,55].

### 1.3. Mental Health and Dispositional Hope

Dispositional hope has been identified as a protective factor against mental health challenges [56]. An optimistic outlook and hopeful drive can bolster resilience, instill a sense of purpose and direction, and foster adaptive coping mechanisms in the face of adversity or difficult life circumstances [56]. Individuals with elevated levels of dispositional hope are more inclined to employ problem-solving techniques, seek out social support, and embrace positive strategies to manage stress, all of which can lead to improved mental health outcomes [57]. Similarly, individuals with heightened levels of dispositional hope typically experience better mental health outcomes, such as decreased psychological distress, lower risk of mental disorders, and enhanced overall well-being [16,58]. Conversely, diminished levels of dispositional hope are linked to heightened vulnerability to mental health issues like depression, anxiety, and hopelessness [56,59,60]. A deficiency in dispositional hope may foster feelings of helplessness, reduce motivation, and cultivate a pessimistic outlook, all of which can impede effective coping and exacerbate mental health challenges.

### 1.4. The Mediating Role of Mental Health in the Relationship between Well-Being and Dispositional Hope

The current study posits that mental health (in terms of the absence of anxiety symptoms) acts as a mediator in the relationship between well-being and dispositional hope. Previous research indicates that higher levels of well-being correlate with improved mental health outcomes, including reduced symptoms of depression and anxiety, greater resilience, and enhanced overall psychological well-being [18,41,61]. These outcomes may facilitate the development of dispositional hope. In this context, positive experiences of well-being—such as feeling positive emotions, having a sense of purpose and meaning, and engaging in positive social relationships—can contribute to better mental health outcomes. Therefore, positive emotions and a sense of purpose may boost psychological resilience, encourage adaptive coping strategies, and foster the cultivation of positive self-beliefs and expectations [44,48].

Individuals with better mental health may possess the cognitive and emotional resources necessary to establish and pursue meaningful goals, maintain positive expectations, and persevere in the face of challenges [23]. In turn, poor mental health—such as heightened psychological distress, symptoms of mental disorders, or impaired functioning—can impede the association between well-being and dispositional hope. Consequently, mental health difficulties may diminish positive well-being experiences, erode hope, and hinder the ability to set and achieve meaningful goals [62].

The relationship between psychological well-being and dispositional hope suggests that individuals with higher psychological well-being are more likely to exhibit elevated levels of dispositional hope [16,22,63]. Thus, we propose that mental health can serve as a mediator in the relationship between psychological well-being and dispositional hope. For instance, individuals with higher levels of psychological well-being tend to experience better mental health, enabling them to maintain a positive outlook, set and pursue goals, and exhibit dispositional hope. Conversely, individuals with poor mental health may struggle with psychological well-being, manifesting symptoms such as depression, anxiety, or low self-esteem [64].

Similarly, higher levels of hedonic well-being are generally linked with improved mental health outcomes. Individuals experiencing greater pleasure, happiness, and life satisfaction typically exhibit better emotional well-being, lower levels of psychological distress, and overall positive mental health. These positive mental health outcomes may facilitate the development and maintenance of dispositional hope [1].

Social well-being is also associated with positive mental health outcomes, as individuals with good mental health often have more fulfilling social relationships, higher levels of social support, and a stronger sense of belonging [65]. In turn, positive social relationships can offer emotional support, encouragement, and resources to pursue goals, thus enhancing mental health and, consequently, increasing dispositional hope [66]. Conversely, individuals experiencing difficulties in forming and maintaining social connections may exhibit poor mental health [67].

### 1.5. The Present Study

The present study aims to examine (a) the association of hedonic, psychological, and social well-being with the mental health (in terms of absence of anxiety symptoms) and dispositional hope of Portuguese citizens, and (b) whether mental health plays a mediating role. Based on a literature review, we propose the following hypotheses:

 **Hypothesis 1:**  *Hedonic well-being, psychological well-being, and social well-being are positively and significantly related to dispositional hope.*

 **Hypothesis 2:**  *Hedonic well-being, psychological well-being, and social well-being* *are positively and significantly related to mental health.*

 **Hypothesis 3:**  *Mental health is positively and significantly related to dispositional hope.*

 **Hypothesis 4:**  *Mental health mediates the positive relationship between dispositional hope and hedonic well-being, psychological well-being, and social well-being.*

## 2. Materials and Methods

### 2.1. Study Design and Participants

The study followed a descriptive correlational, cross-sectional design. The aim was to describe a relationship among variables, without attempting to infer causality. Using non-probabilistic sampling, potential participants had to meet the following criteria: (a) 18 years old and older; (b) reside in Portugal; and (c) able to read and understand Portuguese. The minimum sample size (n = 385) was calculated considering the most conservative scenario (a proportion of 50%), a level of confidence of 95%, and an error margin of 5%. This conservative scenario reduces the possibility of an underpowered study for the true effect size (such as a safeguard power analysis) [68]. No individuals declined participation or failed to finish the survey because of its design, which demanded all questions be answered.

### 2.2. Data Collection Procedures

Data collection took place through an online snowball survey using Google Forms promoted through social media (Facebook, Instagram, and WhatsApp), during the last quarter of 2022. This approach to data collection was chosen due to restrictions resulting from public health measures during the COVID-19 pandemic. The survey form was developed according to the Checklist for Reporting Results of Internet e-Surveys (CHERRIES) guidelines [69]. The researchers shared the survey link on their personal and academic social networking sites to maximize the number of prospective participants. The responses to the questionnaire were automatically collected into an EXCEL spreadsheet for further data analysis.

### 2.3. Measures

**Control Variables**. Gender, age, education, and employment status have been linked to mental health and dispositional hope [23,70,71]. Consequently, we examined whether they needed to be included in our model. In the current study, we accounted for participants’ gender, education, and employment status, factors that are correlated with our outcome variables, as suggested by Becker et al. [72].

**Well-being (Psychological, Emotional [Hedonic], and Social).** Well-being was measured using the Mental Health Continuum-Short Form (MHC-SF; Keyes et al. [73], Portuguese version Matos et al. [74]). This measure provides self-reported scores for the three types of well-being: psychological (PWB) (six items), emotional (EWB) (three items, reflects hedonic well-being), and social well-being (SWB) (five items). Participants indicate the frequency of subjective well-being in the last week, using a scale from 1 (never) to 6 (always). Higher scores indicate greater levels of positive well-being. The MHC-SF has demonstrated good reliability and validity, with reported Cronbach’s alphas ranging from 0.85 to 0.89.

**Dispositional Hope Scale** (Snyder [75]; and validated for European Portuguese by Marques et al. [76]). This eight-item scale assesses dispositional hope and is subdivided into a four-item Agency subscale and a four-item Pathways subscale. Responses are given on a 6-point Likert-type scale (0 = strongly disagree; 5 = strongly agree). An example item is “I can achieve the goals I set myself”. Higher scores on the questionnaire indicate a higher level of dispositional hope. In this study, the Cronbach alpha of the entire scale was 0.89.

**Mental Health.** GAD-7 is a common screening tool for assessing the presence or absence of generalized anxiety disorder, one of the most common mental disorders (Spitzer et al. [77]). This scale was validated for the Portuguese population by Sousa et al. [78]. Responses are provided on a 4-point Likert scale (0 = not at all; 3 = nearly every day). Participants are asked to think about the following aspects of their mental health over the preceding two weeks: “Nervousness or anxiousness”; Inability to stop worrying”; Worrying too much”; “Having trouble relaxing”; “Restlessness”; “Irritability”; and “Feeling afraid” [77]. The final GAD-7 score is calculated by reversing responses, where a higher score of GAD-7 therefore indicates a better mental health status. The Cronbach alpha for this scale was 0.91.

### 2.4. Data Analysis

A sufficient sample size is crucial for detecting any relationship between variables [79]. To conduct mediation analysis, a sample size ranging from 115 to 285 people is enough to detect an indirect influence among the variables under study [79].

The initial analysis involved verifying the assumptions of normality and homoscedasticity using the Kolmogorov–Smirnov test and residual graphics, respectively. Descriptive statistics (i.e., means, standard deviations, percentages, and frequencies) were computed for all measurements and Spearman’s correlation analysis was conducted to evaluate the associations between variables, using IBM SPSS Statistics (version 27). Reliability was assessed with Cronbach’s alpha. We performed a confirmatory factor analysis (CFA) using AMOS 27 to assess our measurement model’s fit. Model fit is considered acceptable when the TLI (Tucker–Lewis Index) and CFI (Comparative Fit Index) values are equal to or greater than 0.90 [80]. A good fit is also indicated when the SRMR (Standardized Root-Mean-square error Residual) and RMSEA (Root Mean Square Error of Approximation) values are equal to or less than 0.05 [81].

We employed the PROCESS macro for SPSS (model 4: mediation analysis [82]) to evaluate our model. For all the analyses, we computed 95% confidence intervals (CIs) for the hypothesized effects with a bootstrapping sample size of 10,000 [83]. Mediation was deemed significant when the 95% confidence interval (CI) of the mediator’s coefficient did not encompass zero. Additionally, bootstrapping approaches have numerous advantageous characteristics, including robustness and accuracy, which make them especially valuable when examining indirect effects [83]. In addition, as proposed by Olvera Astivia and Kroc [84], the predictors were mean-centered. Covariates were included in the mediation analysis to account for possible confounders. This entailed using statistical techniques to account for the impact of these variables and to separate the direct effect of the independent variable on the dependent variable from the indirect effect through the mediator.

### 2.5. Ethical Considerations

The current study complies with the EU General Data Protection Regulation (GDPR2016/679), and the principles of the Helsinki Declaration. Ethical review and approval were obtained by the local ethics committee (CE/02/2022). By accessing the online questionnaire, each participant was informed of the study’s objectives and the anonymous, confidential, and voluntary nature of participation, as well as the possibility of terminating the completion of the questionnaire at any time. Participants who agreed to participate in the study submitted an informed consent form by selecting the “Yes, I Agree” option on the online form instead of the “No thanks” option. Participation was voluntary and uncompensated. To prevent multiple entries from the same computer, only one survey using the same IP address was allowed. In addition, the data collected from the online surveys were exclusively accessible to the researcher and were securely stored in a personal laptop protected by a password.

## 3. Results

### 3.1. Sample Description

The sample of 471 participants consisted of full-time or part-time workers (88.6%), pensioners (6.8%), university students (2.5%), and unemployed people (2.1%); it comprised 402 women (85.4%) and 69 men (14.6%). The mean age was 47.72 years (SD = 11.86). In terms of education, 0.2% had completed the second cycle, 0.2% had completed the third cycle, 5.7% had completed secondary school, 50.6% had completed a bachelor’s degree, and 43.3% had completed a master’s or doctoral degree. The demographic characteristics of the sample reflect the general population of Portuguese adults in terms of age and education.

Table 1 contains the descriptive statistics and correlations between the variables in the study, including means, standard deviations, and Spearman’s correlation coefficients.

Correlation analysis of the three main variables (well-being, mental health, and dispositional hope) showed that mental health and dispositional hope were significantly and positively correlated (r = 0.61, *p* < 0.01), and both were also significantly and positively correlated with the different domains of well-being.

### 3.2. Confirmatory Factor Analysis

Table 2 shows the goodness of fit indices for the hypothesized and alternative models and confirms that the hypothesized model has the best fit.

Our theoretical five-factor model was compared with a series of nested models. The hypothesized five-factor model was the best-fitting model (χ^2^(362) = 1806.29; CFI = 0.91; TLI = 0.91; RMSEA = 0.05; and SRMR = 0.05). The five constructs were treated separately in subsequent statistical tests of our hypotheses.

### 3.3. Mediating Analysis

**Figure 2:**
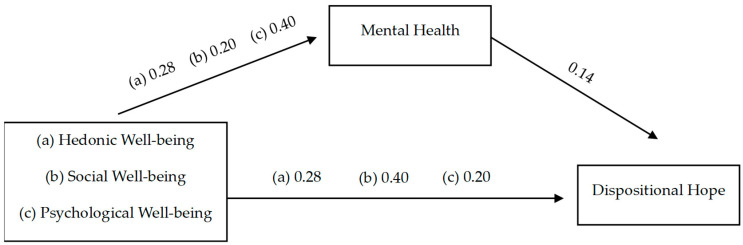
Study model (N = 471).

Figure 2 summarizes the hierarchical multiple regression analyses conducted to examine the indirect effects of well-being on dispositional hope.

In support of hypothesis 1 (a, b, and c), hedonic well-being (=0.28; 95% CI [0.24, 0.34]), social well-being (β = 0.40; 95% CI [0.35, 0.45]), and psychological well-being (β = 0.20; 95% CI [0.15, 0.24]) were positively and significantly related to dispositional hope (*p* < 0.01).

Hypothesis 2 (a, b, and c) postulated a positive relationship between well-being and mental health. Our results support this hypothesis: hedonic well-being (β = 0.28; 95% CI [0.24, 0.34]), social well-being (β = 0.20; 95% CI [0.15, 0.24]), and psychological well-being (β = 0.40; 95% CI [0.35, 0.45]) were all significantly related to mental health (*p* < 0.01).

Hypothesis 3 was also supported: mental health presented a significant and positive relationship with dispositional hope (β = 0.14; 95% CI [0.05, 0.22]).

We conducted a mediation analysis to examine whether mental health mediates the relationship between well-being and dispositional hope (hypothesis 4). The indirect effects for hedonic well-being (β = 0.05; 95% CI [0.008, 0.092]), psychological well-being (β = 0.04; 95% CI [0.007, 0.079]), and social well-being (β = 0.03; 95% CI [0.006, 0.052]) were all significant, indicating a mediating effect.

Table 3 shows the path coefficients for the mediation analysis, confirming the mediating role of mental health in the relationship between well-being and dispositional hope. The mediation effect accounts for 16.0% of the total effect.

## 4. Discussion

This study found that mental health [based on the absence of anxiety symptoms] mediates the relationship between well-being and dispositional hope. All dimensions of well-being showed significant positive associations with dispositional hope and mental health. Such mediation suggests that the impact of well-being on dispositional hope is, at least partially, explained by its influence on mental health. Therefore, enhancing mental health may serve as a pathway through which well-being fosters dispositional hope. Furthermore, as hypothesized, our findings revealed significant positive associations between dispositional hope and hedonic, psychological, and social well-being. Our findings are consistent with previous research [22,85] showing that positive emotions and social connections are critical for promoting hope. Moreover, our results showed that individuals with elevated levels of hedonic, psychological, and social well-being are more likely to encounter better mental health outcomes [58]. This underscores the importance of promoting positive emotions, personal development, and social connections to enhance mental well-being.

This study makes a unique contribution to the literature by demonstrating the mediating role of mental health in the relationship between various dimensions of well-being and dispositional hope. The study strengthens conceptual frameworks that underscore the interconnections among these constructs, highlighting the significance of considering multiple facets of well-being and their influence on mental health and dispositional hope [86]. Furthermore, these results underscore the importance of positive emotions, personal development, social connections, and mental well-being in nurturing a hopeful perspective. Despite resilience and optimism not being within the scope of this research, our study aligns with previous studies that regard well-being and mental health as pivotal components in their fostering [87].

Moreover, the implications of this study extend to practical interventions and programs aimed at promoting well-being and mental health. They suggest enhancing hedonic, psychological, and social well-being can yield positive outcomes for an individual’s mental health and dispositional hope. Interventions such as mindfulness training, cognitive behavioral therapy, and community-building activities can be effective in improving mental well-being and social relationships [58]. Furthermore, the results emphasize the need for a comprehensive approach that considers the intricate interplay between well-being, mental health, and dispositional hope when formulating interventions and policies aimed at enhancing positive mental health literacy [88], overall well-being, and resilience. This comprehensive approach should combine mental health support with social engagement and personal development activities tailored to the needs of different population groups.

### Limitations and Future Research Avenues

Despite this study’s significant findings and contributions, it has several limitations that suggest areas for improvement and avenues for future research. Firstly, the cross-sectional design limits our ability to infer causality. Longitudinal studies could shed light on how changes in well-being over time affect mental health and hope. Secondly, while self-assessments are prone to bias, we used validated scales to increase reliability. Future research could benefit from employing a multi-method approach that integrates self-report measures with objective indicators or behavioral observations to enhance the validity of the findings. Thirdly, the sample size was relatively small with a high percentage of women, which determine an under-representation of wider population. Future research should aim to include larger and more diverse samples. Fourthly, the web survey methodology may increase the risk of biased data, namely due to the digital divide and self-selection bias. Fifthly, mental health status was only assessed through the presence or absence of anxiety symptoms, excluding a large range of other mental health conditions. This limitation elicits the need for caution when interpreting the results of this study. In addition, and given that physical and mental health are interconnected, we suggest that further studies use measures that evaluate positive mental health as well as physical health indicators. Finally, although this study identified relationships between well-being, mental health, and dispositional hope, it did not fully explore the directionality of these relationships. It is plausible that the relationships could be bidirectional or reciprocal. Future studies could use lagged panel designs to examine whether well-being predicts future mental health and dispositional hope or vice versa.

Further research could delve into the underlying mechanisms and processes through which well-being dimensions influence mental health and dispositional hope. For instance, exploring the role of coping strategies, self-efficacy, resilience, or social support as potential mediators or moderators could enhance our understanding of the pathways involved. Investigating the role of coping strategies, such as problem- or emotion-focused coping, could provide deeper insights into the influence of well-being on mental health and hope. Additionally, investigating the influence of cultural and contextual factors on the relationships between well-being, mental health, and dispositional hope could be relevant, as cultural variations in the conceptualization and manifestation of these constructs may impact the proposed relationships. Future research could examine how cultural attitudes toward mental health influence the relationship between well-being and dispositional hope. Comparative studies across different cultural contexts could reveal how social norms and values influence the interplay between well-being, mental health, and dispositional hope. Future research could also focus on developing and evaluating interventions that simultaneously promote well-being, mental health, and dispositional hope. Developing multifaceted interventions that simultaneously target hedonic, psychological, and social well-being could show synergistic effects on mental health and hope. Specifically, implementing and assessing the effectiveness of interventions targeting multiple dimensions could provide valuable insights into potential synergistic effects and strategies for enhancing overall well-being and hopefulness.

By addressing these limitations and pursuing these avenues of research, scholars can further advance our understanding of the intricate relationships between well-being, mental health, dispositional hope, and the underlying processes. For instance, several structural factors such as poverty and political despair may decrease overall well-being and hope [89]. These considerations regarding context should prompt us to examine the complex interactions between individuals, groups, and society in a non-linear manner [90]. This knowledge can inform the development of evidence-based interventions and policies aimed at promoting holistic well-being and psychological resilience across diverse populations.

## 5. Conclusions

This study offers convincing evidence supporting the positive and significant relationships between dispositional hope, mental health, and hedonic, psychological, and social well-being. Findings suggest that enhancing well-being can improve mental health and promote hope. The theoretical and practical implications outlined here can provide direction for future research and interventions geared toward fostering well-being and favorable psychological outcomes.

## Figures and Tables

**Table 1:** Descriptive statistics and correlations among study variables (N = 471).

	Mean	SD	1	2	3	4	5	6	7	8	9
1. Level of Education ^a^	7.28	0.82	-								
2. Age	47.72	11. 86	−0.12 **	-							
3. Employment Status ^b^	3.99	0.87	−0.09 *	0.07	-						
4. Gender	-	-	0.03	−0.09	−0.10 *	-					
5. Psychological well-being ^c^	4.43	0.99	0.11 *	0.07	−0.07	0.00	-				
6. Hedonic well-being ^c^	4.54	1.06	0.07	−0.01	−0.007	0.00	0.75 **	-			
7. Social well-being ^c^	3.41	1.18	0.13 **	0.05	0.04	0.08 *	0.72 **	0.61 **	-		
8. Mental Health ^d^	2.06	0.65	0.09 *	−0.78	0.12 *	−0.07	0.64 **	0.62 **	0.58 **	-	
9. Dispositional Hope ^c^	3.83	0.65	0.12 **	0.11 *	0.02	0.01	0.51 **	0.60 **	0.52 **	0.61 **	-

Notes: correlations between the sociodemographic variables (except age) and the variables under study were evaluated using the Spearman’s Rho correlation coefficient. ^a^ Education (1 = primary education not completed; 2 = primary education; 3 = sixth grade; 4 = ninth grade; 5 = high school; 6 = undergraduate degree; and 7 = master degree or Ph.D.); ^b^ Employment Status (1 = student; 2 = housework; 3 = part-time job; 4 = full-time job; 5 = unemployed; and 6 = retired); ^c^ 6-point scale; ^d^ 4-point scale; * *p* < 0.05 and ** *p* < 0.01, all two-tailed tests.

**Table 2:** CFAs for the hypothesized and alternative models.

	*df*	*X* ^2^	*X*^2^ Difference	CFI	TLI	RMSEA	SRMR
Five-factor model	359	1,806,287 **	327.153 **	0.91	0.91	0.05	0.05
Three-factor model ^a^	374	2,694,178 **	887.891 **	0.77	0.76	0.09	0.10
Two-factor model ^b^	376	2,744,005 **	937.718 **	0.77	0.76	0.09	0.11
One-factor model ^c^	379	4,343,412 **	2537.125 **	0.61	0.58	0.12	0.13

Notes. ^a^ Merge psychological, emotional, and social well-being; ^b^ merge psychological, emotional, social well-being, and mental health; ^c^ merge psychological, emotional, social well-being, mental health, and dispositional hope. CFAs = confirmatory factor analyses; *df* = degrees of freedom; CFI = comparative fit index; TLI = Tucker–Lewis index; RMSEA = root mean square error of approximation; SRMR = standardized root-mean-square error residual; and ** *p* < 0.01.

**Table 3:** Mediating effect analysis.

Predictors	Mediator			Outcome		
	Mental Health			Dispositional Hope		
	β	SE(β)	95% CI	β	SE(β)	95% CI
*Control Variables*						
Educational level	0.08	0.03	[0.02, 0.15]	0.05	0.03	[−0.00, 0.11]
Age	0.01	0.00	[0.00, 0.01]	0.00	0.00	[−0.00, 0.01]
Employment status	−0.01	0.04	[−0.09, 0.06]	−0.03	0.03	[−0.09, 0.04]
*Main effects*						
Psychological well-being	0.40	0.03	[0.35, 0.45]	0.40	0.03	[0.35, 0.45]
Hedonic well-being	0.28	0.02	[0.24, 0.34]	0.30	0.03	[0.25, 0.36]
Social well-being	0.20	0.02	[0.15, 0.24]	0.19	0.02	[0.15, 0.24]
*Mediator*						
Mental Health	-	-	-	0.14	0.05	[0.05, 0.22]
R^2^		0.20 **			0.16 **	

Regression coefficient estimates (β), standard error (SE), 95% confidence intervals (CIs), ** *p* < 0.01.

## Data Availability

The data are available upon reasonable request.

## References

[1] Keyes C.L. (2006). Subjective well-being in mental health and human development research worldwide: An introduction. Soc. Indic. Res..

[2] Das K.V., Jones-Harrell C., Fan Y., Ramaswami A., Orlove B., Botchwey N. (2020). Understanding subjective well-being: Perspectives from psychology and public health. Public Health Rev..

[3] Costanza R., Daly L., Fioramonti L., Giovannini E., Kubiszewski I., Mortensen L.F., Pickett K.E., Ragnarsdottir K.V., De Vogli R., Wilkinson R. (2016). Modelling and measuring sustainable wellbeing in connection with the UN Sustainable Development Goals. Ecol. Econ..

[4] Judge T.A., Kammeyer-Mueller J.D. (2011). Happiness as a societal value. Acad. Manag. Perspect..

[5] Joshanloo M. (2016). Revisiting the empirical distinction between hedonic and eudaimonic aspects of well-being using exploratory structural equation modeling. J. Happiness Stud. Interdiscip. Forum Subj. Well-Being.

[6] Keyes C.L. (2002). The mental health continuum: From languishing to flourishing in life. J. Health Soc. Behav..

[7] Ryan R.M., Deci E.L. (2001). On happiness and human potentials: A review of research on hedonic and eudaimonic well-being. Annu. Rev. Psychol..

[8] Chen H., Zeng Z. (2022). Associations of hedonic and eudaimonic orientations with subjective experience and objective functioning in academic settings: The mediating roles of academic behavioral engagement and procrastination. Front. Psychol..

[9] Extremera N., Ruiz-Aranda D., Pineda-Galán C., Salguero J.M. (2011). Emotional intelligence and its relation with hedonic and eudaimonic well-being: A prospective study. Personal. Individ. Differ..

[10] King L.A., Sheldon K.M., Kashdan T.B., Steger M.F. (2011). Are we there yet? What happened on the way to the demise of positive psychology. Designing Positive Psychology: Taking Stock and Moving Forward.

[11] Keyes C.L. (1998). Social well-being. Soc. Psychol. Q..

[12] Mosley-Johnson E., Garacci E., Wagner N., Mendez C., Williams J.S., Egede L.E. (2019). Assessing the relationship between adverse childhood experiences and life satisfaction, psychological well-being, and social well-being: United States Longitudinal Cohort 1995–2014. Qual. Life Res..

[13] Brown N.J., Rohrer J.M. (2020). Easy as (happiness) pie? A critical evaluation of a popular model of the determinants of well-being. J. Happiness Stud. Interdiscip. Forum Subj. Well-Being.

[14] Huppert F.A. (2009). Psychological well-being: Evidence regarding its causes and consequences. Appl. Psychol. Health Well-Being.

[15] Park H.J., Jeong D.Y. (2015). Psychological well-being, life satisfaction, and self-esteem among adaptive perfectionists, maladaptive perfectionists, and nonperfectionists. Personal. Individ. Differ..

[16] Yıldırım M., Arslan G. (2022). Exploring the associations between resilience, dispositional hope, preventive behaviours, subjective well-being, and psychological health among adults during early stage of COVID-19. Curr. Psychol..

[17] Snyder C.R., Shorey H.S., Cheavens J., Pulvers K.M., Adams V.H., Wiklund C. (2002). Hope and academic success in college. J. Educ. Psychol..

[18] Ruggeri K., Garcia-Garzon E., Maguire Á., Matz S., Huppert F.A. (2020). Well-being is more than happiness and life satisfaction: A multidimensional analysis of 21 countries. Health Qual. Life Outcomes.

[19] Simões A.V. (2023). Street Art in Aveiro: City Walls as Dialogic Spaces of Collective Memories and Identity. Societies.

[20] Cheavens J.S., Feldman D.B., Gum A., Michael S.T., Snyder C.R. (2006). Hope therapy in a community sample: A pilot investigation. Soc. Indic. Res..

[21] Feldman D.B., Dreher D.E. (2012). Can hope be changed in 90 minutes? Testing the efficacy of a single-session goal-pursuit intervention for college students. J. Happiness Stud..

[22] Murphy E.R. (2023). Hope and well-being. Curr. Opin. Psychol..

[23] Snyder C.R. (2002). Hope theory: Rainbows in the mind. Psychol. Inq..

[24] Espinoza M., Molinari G., Etchemendy E., Herrero R., Botella C., Baños Rivera R.M. (2017). Understanding dispositional hope in general and clinical populations. Appl. Res. Qual. Life.

[25] Karataş Z., Uzun K., Tagay Ö. (2021). Relationships Between the Life Satisfaction, Meaning in Life, Hope and COVID-19 Fear for Turkish Adults During the COVID-19 Outbreak. Front. Psychol..

[26] Chan S., Fung C., Huang Q. (2023). Positive Emotions, Hope, and Life Satisfaction in Chinese Older Adults: An Application of Broaden-and-Build Model. Int. J. Aging Hum. Dev..

[27] Feldman D.B., Rand K.L., Kahle-Wrobleski K. (2009). Hope and goal attainment: Testing a basic prediction of hope theory. J. Soc. Clin. Psychol..

[28] Caprara M., Gerbino M., Mebane M.E., Ramirez-Uclés I.M. (2022). Self-efficacy beliefs in managing positive emotions: Associations with positive affect, negative affect, and life satisfaction across gender and ages. Front. Hum. Neurosci..

[29] Carver C.S., Scheier M.F., Segerstrom S.C. (2010). Optimism. Clin. Psychol. Rev..

[30] Lee J.Y., Gallagher M.W. (2018). Hope and Well-Being. The Oxford Handbook of Hope.

[31] Bareket-Bojmel L., Shahar G., Abu-Kaf S., Margalit M. (2021). Perceived social support, loneliness, and hope during the COVID-19 Pandemic: Testing a mediating model in the UK, USA, and Israel. Br. J. Clin. Psychol..

[32] Haim-Litevsky D., Komemi R., Lipskaya-Velikovsky L. (2023). Sense of Belonging, Meaningful Daily Life Participation, and Well-Being: Integrated Investigation. Int. J. Environ. Res. Public Health.

[33] Gallagher M.W. (2009). Hope and optimism and their role in positive psychological theories of mental health: A systematic review. J. Happiness Stud..

[34] Xiang G., Teng Z., Li Q., Chen H., Guo C. (2020). The influence of perceived social support on hope: A longitudinal study of older-aged adolescents in China. Child. Youth Serv. Rev..

[35] Litwic-Kaminska K., Błachnio A., Kapsa I., Brzeziński Ł., Kopowski J., Stojković M., Hinićm D., Krsmanovićm I., Ragni B., Sulla F. (2023). Resilience, Positivity and Social Support as Perceived Stress Predictors among University Students. Int. J. Environ. Res. Public Health.

[36] Ryff C.D. (1989). Happiness is everything, or is it? Explorations on the meaning of psychological well-being. J. Personal. Soc. Psychol..

[37] Saleem M., Rizvi T., Bashir I. (2023). The role of hope in buffering the influence of intolerance of uncertainty on student’s psychological well-being. Peace Confl. J. Peace Psychol..

[38] Senger A.R. (2023). Hope’s relationship with resilience and mental health during the COVID-19 pandemic. Curr. Opin. Psychol..

[39] Ke G.N., Grajfoner D., Wong R.M., Carter S., Khairudin R., Lau W.Y., Kamal K.A., Lee S.C. (2022). Building the Positive Emotion-Resilience-Coping Efficacy Model for COVID-19 Pandemic. Front. Psychol..

[40] Stephan U. (2018). Entrepreneurs’ mental health and well-being: A review and research agenda. Acad. Manag. Perspect..

[41] Keyes C.L. (2005). Mental illness and/or mental health? Investigating axioms of the complete state model of health. J. Consult. Clin. Psychol..

[42] Lamers S.M., Westerhof G.J., Bohlmeijer E.T., ten Klooster P.M., Keyes C.L. (2011). Evaluating the psychometric properties of the Mental Health Continuum-Short Form (MHC-SF). J. Clin. Psychol..

[43] World Health Organization (2014). Mental Health: A State of Well-Being.

[44] Ryff C.D. (2014). Psychological well-being revisited: Advances in the science and practice of eudaimonia. Psychother. Psychosom..

[45] Diener E., Wirtz D., Tov W., Kim-Prieto C., Choi D., Oishi S., Biswas-Diener R. (2010). New well-being measures: Short scales to assess flourishing and positive and negative feelings. Soc. Indic. Res..

[46] Huppert F.A., So T.T. (2013). Flourishing across Europe: Application of a new conceptual framework for defining well-being. Soc. Indic. Res..

[47] Lyubomirsky S., King L., Diener E. (2005). The benefits of frequent positive affect: Does happiness lead to success?. Psychol. Bull..

[48] Fredrickson B.L. (2001). The role of positive emotions in positive psychology: The broaden-and-build theory of positive emotions. Am. Psychol..

[49] Diener E. (2000). Subjective well-being: The science of happiness and a proposal for a national index. Am. Psychol..

[50] Kring A.M., Sloan D.M. (2009). Emotion Regulation and Psychopathology: A Transdiagnostic Approach to Etiology and Treatment.

[51] Cruwys T., Haslam S.A., Dingle G.A., Haslam C., Jetten J. (2014). Depression and social identity: An integrative review. Personal. Soc. Psychol. Rev..

[52] Cohen S. (2004). Social relationships and health. Am. Psychol..

[53] Wickramaratne P.J., Yangchen T., Lepow L., Patra B.G., Glicksburg B., Talati A., Adekkanattu P., Ryu E., Biernacka J.M., Charney A. (2022). Social connectedness as a determinant of mental health: A scoping review. PLoS ONE.

[54] Cacioppo J.T., Hughes M.E., Waite L.J., Hawkley L.C., Thisted R.A. (2006). Loneliness as a specific risk factor for depressive symptoms: Cross-sectional and longitudinal analyses. Psychol. Aging.

[55] Holt-Lunstad J., Smith T.B., Layton J.B. (2010). Social relationships and mortality risk: A meta-analytic review. PLoS Med..

[56] Feldman D.B., Snyder C.R. (2005). Hope and the meaningful life: Theoretical and empirical associations between goal-directed thinking and life meaning. J. Soc. Clin. Psychol..

[57] Smithson M., Shou Y., Dawel A., Calear A.L., Farrer L., Cherbuin N. (2022). The Psychological Benefits of an Uncertain World: Hope and Optimism in the Face of Existential Threat. Front. Psychol..

[58] Laranjeira C., Querido A. (2022). Hope and Optimism as an Opportunity to Improve the “Positive Mental Health” Demand. Front. Psychol..

[59] Lopez S.J., Ciarlelli R., Coffman L., Stone M., Wyatt L. (2003). Diagnosing for strength or pathology?. J. Posit. Psychol..

[60] Corrigan J.A., Schutte N.S. (2023). The Relationships between the Hope Dimensions of Agency Thinking and Pathways Thinking with Depression and Anxiety: A Meta-Analysis. Int. J. Appl. Posit. Psychol..

[61] Gautam S., Jain A., Chaudhary J., Gautam M., Gaur M., Grover S. (2024). Concept of mental health and mental well-being, it’s determinants and coping strategies. Indian J. Psychiatry.

[62] Slezackova A., Stecz P., Millova K., Krafft A.M., Guse T., Slezackova A. (2023). Hope and Mental Health Among Czech and Polish Adults in a Macrosocial Perspective and Religiosity Context. Hope across Cultures. Cross-Cultural Advancements in Positive Psychology.

[63] Muyan-Yılık M., Demir A. (2020). A pathway towards subjective well-being for Turkish university students: The roles of dispositional hope, cognitive flexibility, and coping strategies. J. Happiness Stud..

[64] Foster K., Roche M., Giandinoto J.A., Furness T. (2020). Workplace stressors, psychological well-being, resilience, and caring behaviours of mental health nurses: A descriptive correlational study. Int. J. Ment. Health Nurs..

[65] Van Lente E., Barry M.M., Molcho M., Morgan K., Watson D., Harrington J., McGee H. (2012). Measuring population mental health and social well-being. Int. J. Public Health.

[66] Merolla A.J., Bernhold Q., Peterson C. (2021). Pathways to connection: An intensive longitudinal examination of state and dispositional hope, day quality, and everyday interpersonal interaction. J. Soc. Pers. Relatsh..

[67] Kawachi I., Berkman L.F. (2001). Social ties and mental health. J. Urban Health.

[68] Perugini M., Gallucci M., Costantini G. (2014). Safeguard power as a protection against imprecise power estimates. Perspect. Psychol. Sci..

[69] Eysenbach G. (2004). Improving the quality of Web surveys: The Checklist for Reporting Results of Internet E-Surveys (CHERRIES). J. Med. Internet Res..

[70] Gerino E., Rollè L., Sechi C., Brustia P. (2017). Loneliness, Resilience, Mental Health, and Quality of Life in Old Age: A Structural Equation Model. Front. Psychol..

[71] Kiely K.M., Brady B., Byles J. (2019). Gender, mental health and ageing. Maturitas.

[72] Becker T.E., Atinc G., Breaugh J.A., Carlson K.D., Edwards J.R., Spector P.E. (2016). Statistical control in correlational studies: 10 essential recommendations for organizational researchers. J. Organ. Behav..

[73] Keyes C.L., Wissing M., Potgieter J., Temane M., Kruger A., van Rooy S. (2008). Evaluation of the Mental Health Continuum—Short Form (MHC-SF) in Swetsana-speaking South Africans. Clin. Psychol. Psychother..

[74] Matos A.P., André R.S., Cherpe S., Rodrigues D., Figueira C., Pinto A.M. (2010). Estudo Psicométrico preliminar da Mental Health Continuum—Short Form—For youth numa amostra de adolescentes portugueses. Psychologica.

[75] Snyder C.R., Harris C., Anderson J.R., Holleran S.A., Irving L.M., Sigmon S.T. (1991). The will and the ways: Development and validation of an individual-differences measure of hope. J. Personal. Soc. Psychol..

[76] Marques S.C., Lopez S.J., Fontaine A.M., Coimbra S., Mitchell J. (2014). Validation of a Portuguese version of the Snyder Hope Scale in a sample of high school students. J. Psychoeduc. Assess..

[77] Spitzer R.L., Kroenke K., Williams J.B., Löwe B. (2006). A brief measure for assessing generalized anxiety disorder: The GAD-7. Arch. Intern. Med..

[78] Sousa T.V., Viveiros V., Chai M.V., Vicente F., Jesus G., Carnot M., Gordo A., Ferreira P. (2015). Reliability and validity of the Portuguese version of the Generalized Anxiety Disorder (GAD-7) scale. Health Qual. Life Outcomes.

[79] Fritz M.S., MacKinnon D.P. (2007). Required sample size to detect the mediated effect. Psychol. Sci..

[80] Marsh H.W., Hau K.-T., Grayson D., Maydeu-Olivares A., McArdle J.J. (2005). Goodness of Fit in Structural Equation Models. Contemporary Psychometrics: A Festschrift for Roderick P. McDonald.

[81] Fabrigar L.R., MacCallum R.C., Wegener D.T., Strahan E.J. (1999). Evaluating the use of exploratory factor analysis in psychological research. Psychol. Methods.

[82] Hayes A.F., Scharkow M. (2013). The relative trustworthiness of inferential tests of the indirect effect in statistical mediation analysis: Does method really matter?. Psychol. Sci..

[83] Preacher K.J., Hayes A.F. (2008). Asymptotic and resampling strategies for assessing and comparing indirect effects in multiple mediator models. Behav. Res. Methods.

[84] Olvera Astivia O.L., Kroc E. (2019). Centering in Multiple Regression does Not Always Reduce Multicollinearity: How to Tell When Your Estimates Will not Benefit from Centering. Educ. Psychol. Meas..

[85] Pleeging E., Burger M., van Exel J. (2021). The relations between hope and subjective well-being: A literature overview and empirical analysis. Appl. Res. Qual. Life.

[86] Shoshani A., Steinmetz S. (2014). Positive psychology at school: A school-based intervention to promote adolescents’ mental health and well-being. J. Happiness Stud..

[87] Sayed T., Malan H., Fourie E. (2024). Exploring the associations between resilience and psychological well-being among South Africans during COVID-19. Front. Psychol..

[88] Carvalho D., Sequeira C., Querido A., Tomás C., Morgado T., Valentim O., Moutinho L., Gomes J., Laranjeira C. (2022). Positive Mental Health Literacy: A Concept Analysis. Front. Psychol..

[89] Bird L., Thomas E., Wenzel M. (2024). We despair: Examining the role of political despair for collective action and well-being. Eur. J. Soc. Psychol..

[90] Bou Zeineddine F., Leach C.W. (2021). Feeling and thought in collective action on social issues: Toward a systems perspective. Soc. Personal. Psychol. Compass.

